# Supplementary figures and images for: Smallpox vaccination induces a substantial increase in commensal skin bacteria that promote pathology and influence the host response
Source: PLoS Pathog. 2022 Apr 21;18(4):e1009854. doi: 10.1371/journal.ppat.1009854 (PMC9022886; doi:10.1371/journal.ppat.1009854)

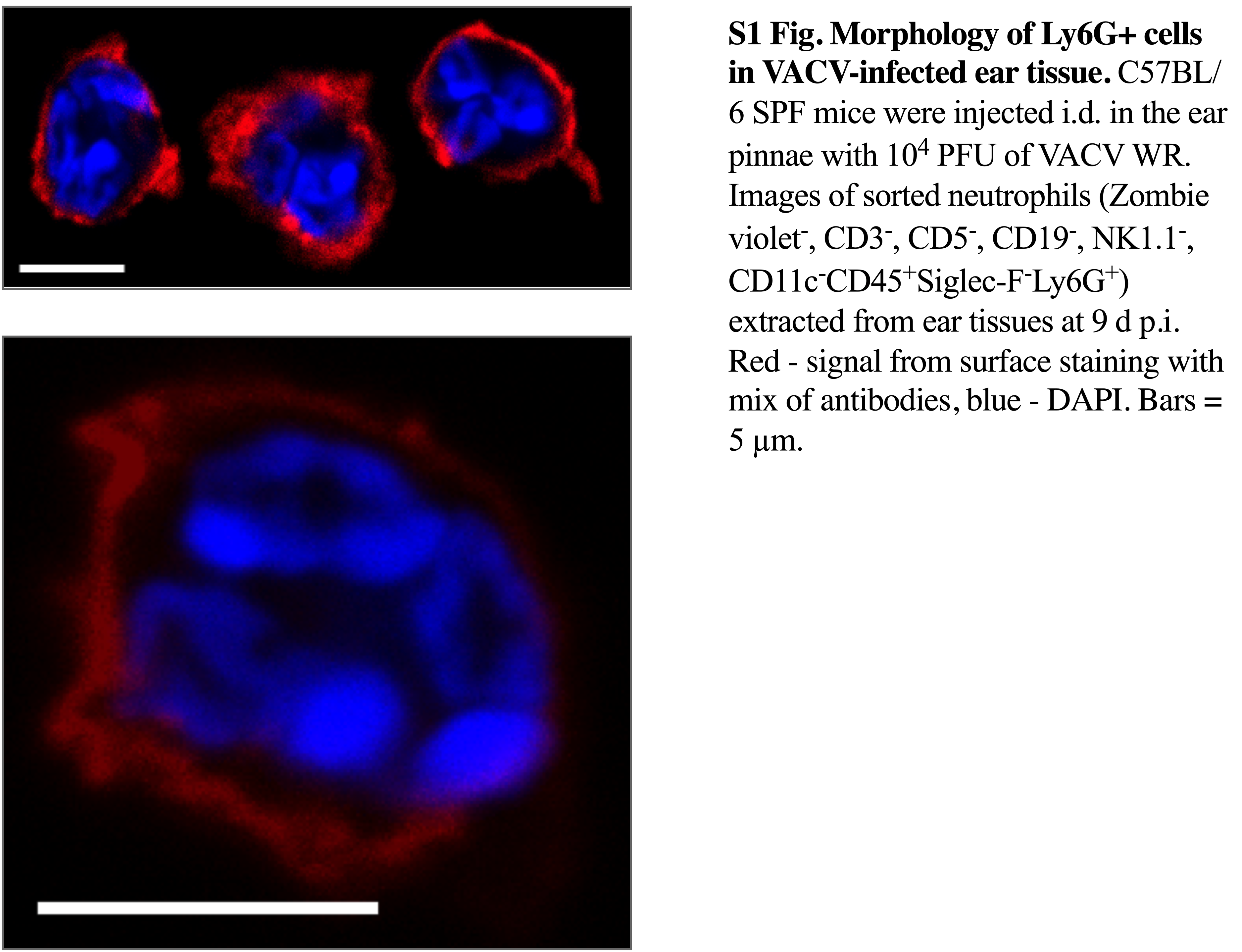

Supplement: S1 Fig — C57BL/6 SPF mice were injected i.d. in the ear pinnae with 104 PFU of VACV WR. Images of sorted neutrophils (Zombie violet-, CD3-, CD5-, CD19-, NK1.1-, CD11c-CD45+Siglec-F-Ly6G+) extracted from ear tissues at 9 d p.i. Red—signal from surface staining with mix of antibodies, blue—DAPI. Bars = 5 μm. (TIFF) [file ppat.1009854.s001.tiff]

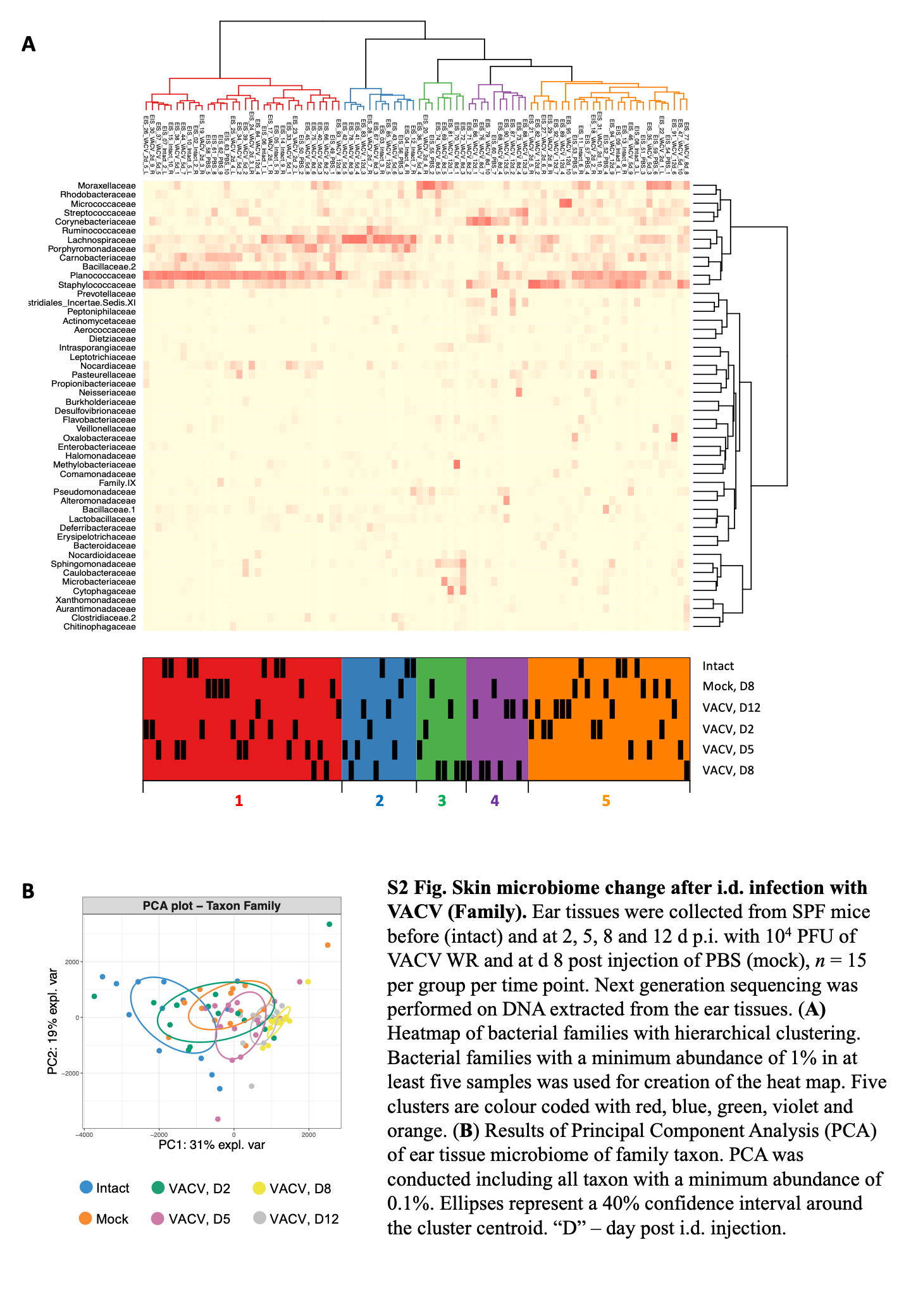

Supplement: S2 Fig — Ear tissues were collected from SPF mice before (intact) and at 2, 5, 8 and 12 d p.i. with 104 PFU of VACV WR and at d 8 post injection of PBS (mock), n = 15 per group per time point. Next generation sequencing was performed on DNA extracted from the ear tissues. (A) Heatmap of bacterial families with hierarchical clustering. Bacterial families with a minimum abundance of 1% in at least five samples was used for creation of the heat map. Five clusters are colour coded with red, blue, green, violet and orange. (B) Results of Principal Component Analysis (PCA) of ear tissue microbiome of family taxon. PCA was conducted including all taxon with a minimum abundance of 0.1%. Ellipses represent a 40% confidence interval around the cluster centroid. “D”–day post i.d. injection. (TIFF) [file ppat.1009854.s002.tiff]

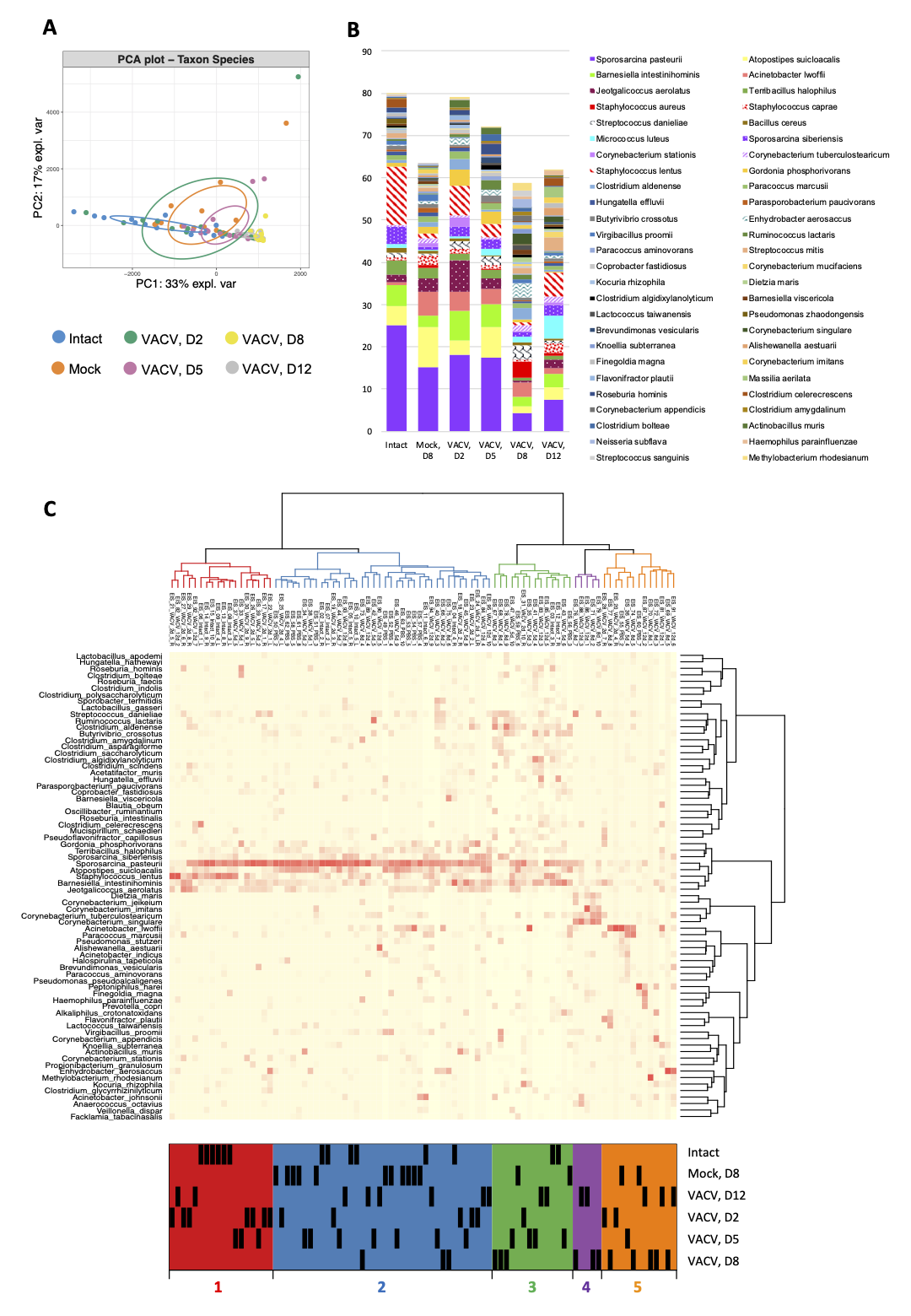

Supplement: S3 Fig — Ear tissues were collected from SPF mice before (intact) and at 2, 5, 8 and 12 d p.i. with 104 PFU of VACV WR and at d 8 after injection of PBS (mock), n = 15 per group per time point. Next generation sequencing was performed on DNA extracted from the ear tissues. (A) Results of Principal Component Analysis (PCA) of ear tissue microbiome of specie taxon. PCA was conducted including all taxon with a minimum abundance of 0.1%. Ellipses represent a 40% confidence interval around the cluster centroid. (B) Relative abundance of most prevalent taxa of ear tissue bacterial species. (C) Heatmap of bacterial species with hierarchical clustering. Bacterial species with a minimum abundance of 1% in at least five samples was used for creation of the heat map. Five clusters are colour coded with red, blue, green, violet and orange. “D”–day post i.d. injection. (TIFF) [file ppat.1009854.s003.tiff]

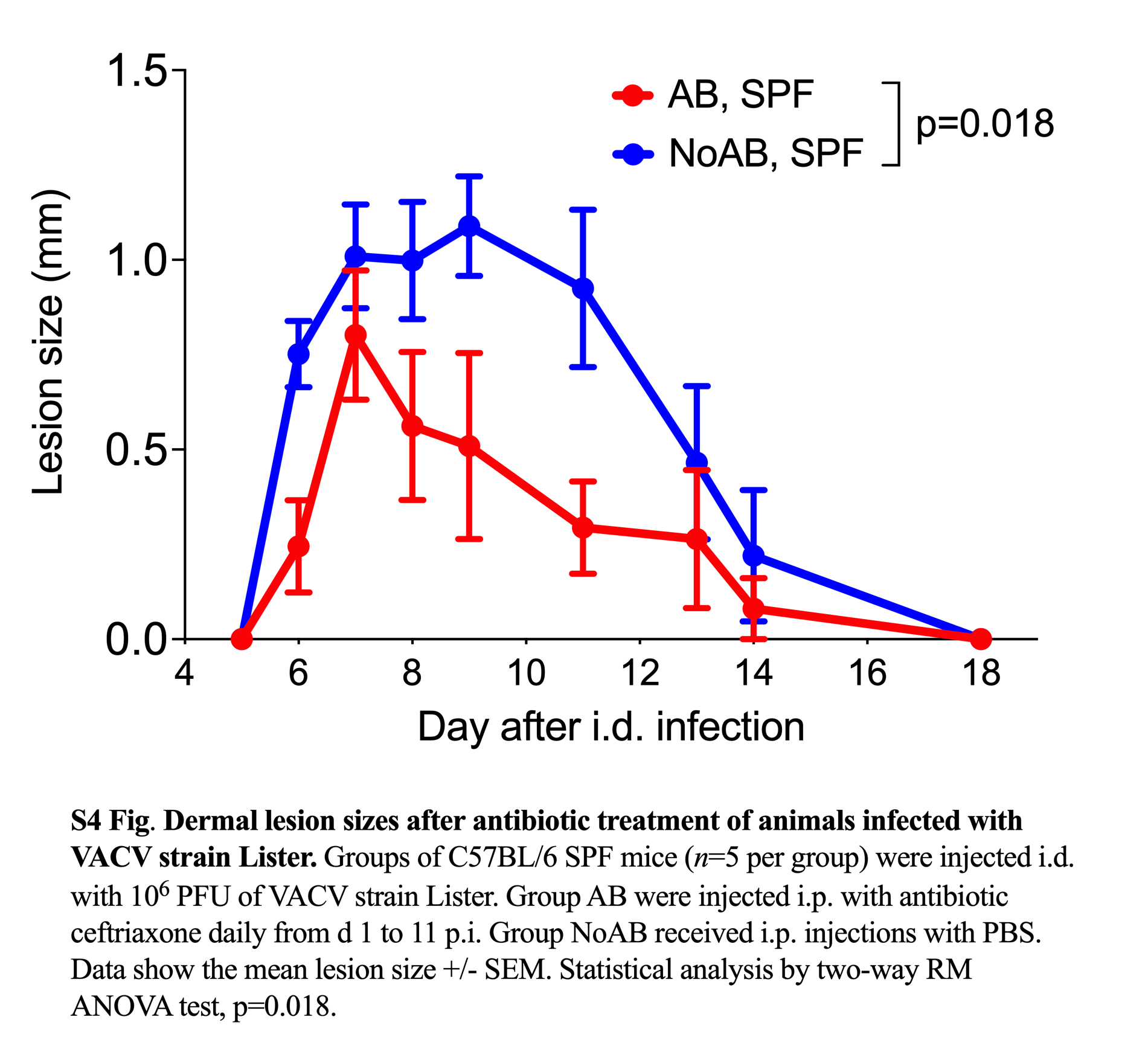

Supplement: S4 Fig — Groups of C57BL/6 SPF mice (n = 5 per group) were injected i.d. with 106 PFU of VACV strain Lister. Group AB were injected i.p. with antibiotic ceftriaxone daily from d 1 to 11 p.i. Group NoAB received i.p. injections with PBS. Data show the mean lesion size +/- SEM. Statistical analysis by two-way RM ANOVA test, p = 0.018. (TIFF) [file ppat.1009854.s004.tiff]

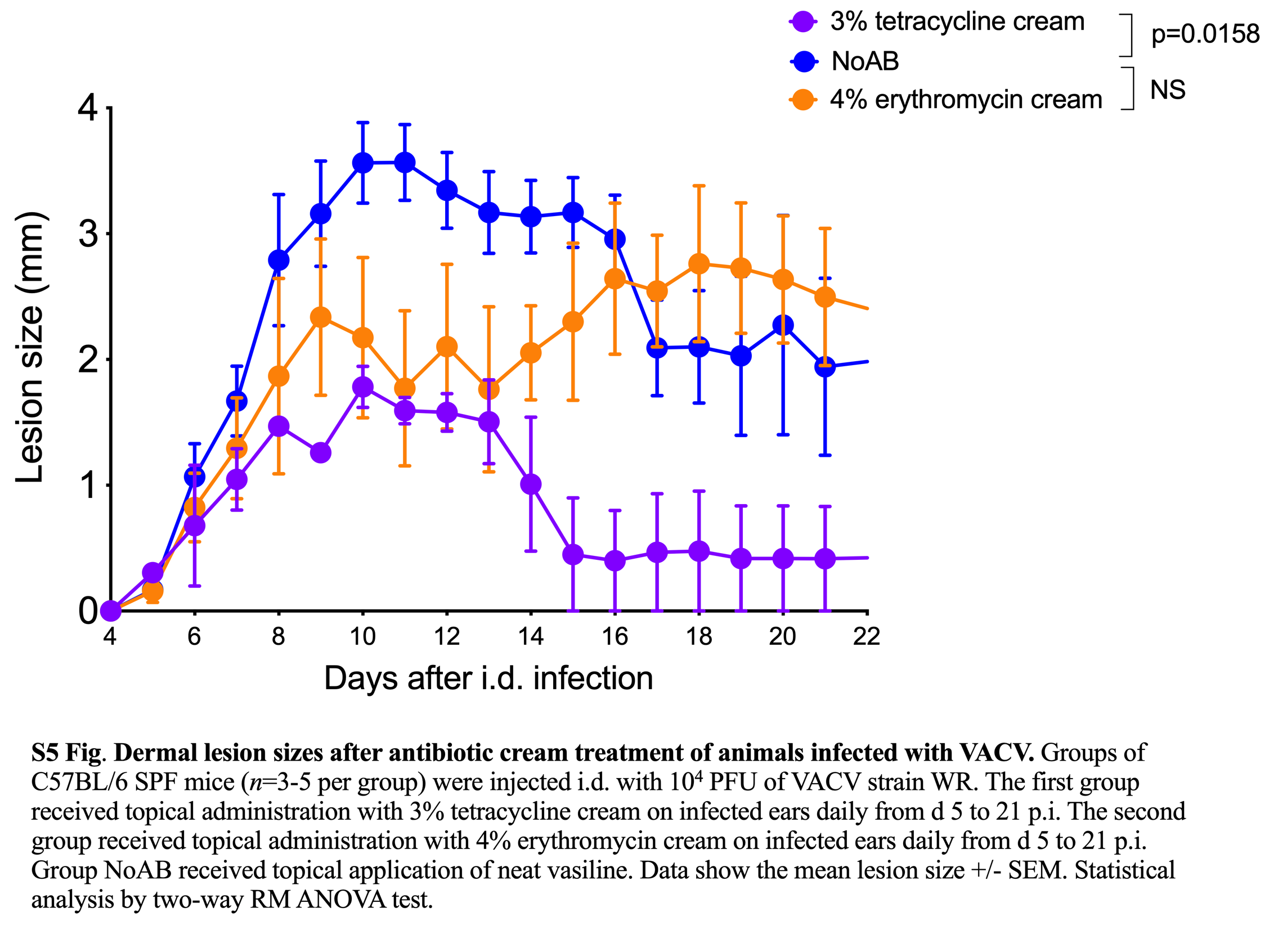

Supplement: S5 Fig — Groups of C57BL/6 SPF mice (n = 3–5 per group) were injected i.d. with 104 PFU of VACV strain WR. The first group received topical administration with 3% tetracycline cream on infected ears daily from d 5 to 21 p.i. The second group received topical administration with 4% erythromycin cream on infected ears daily from d 5 to 21 p.i. Group NoAB received topical application of neat vaseline. Data show the mean lesion size +/- SEM. Statistical analysis by two-way RM ANOVA test. (TIFF) [file ppat.1009854.s005.tiff]

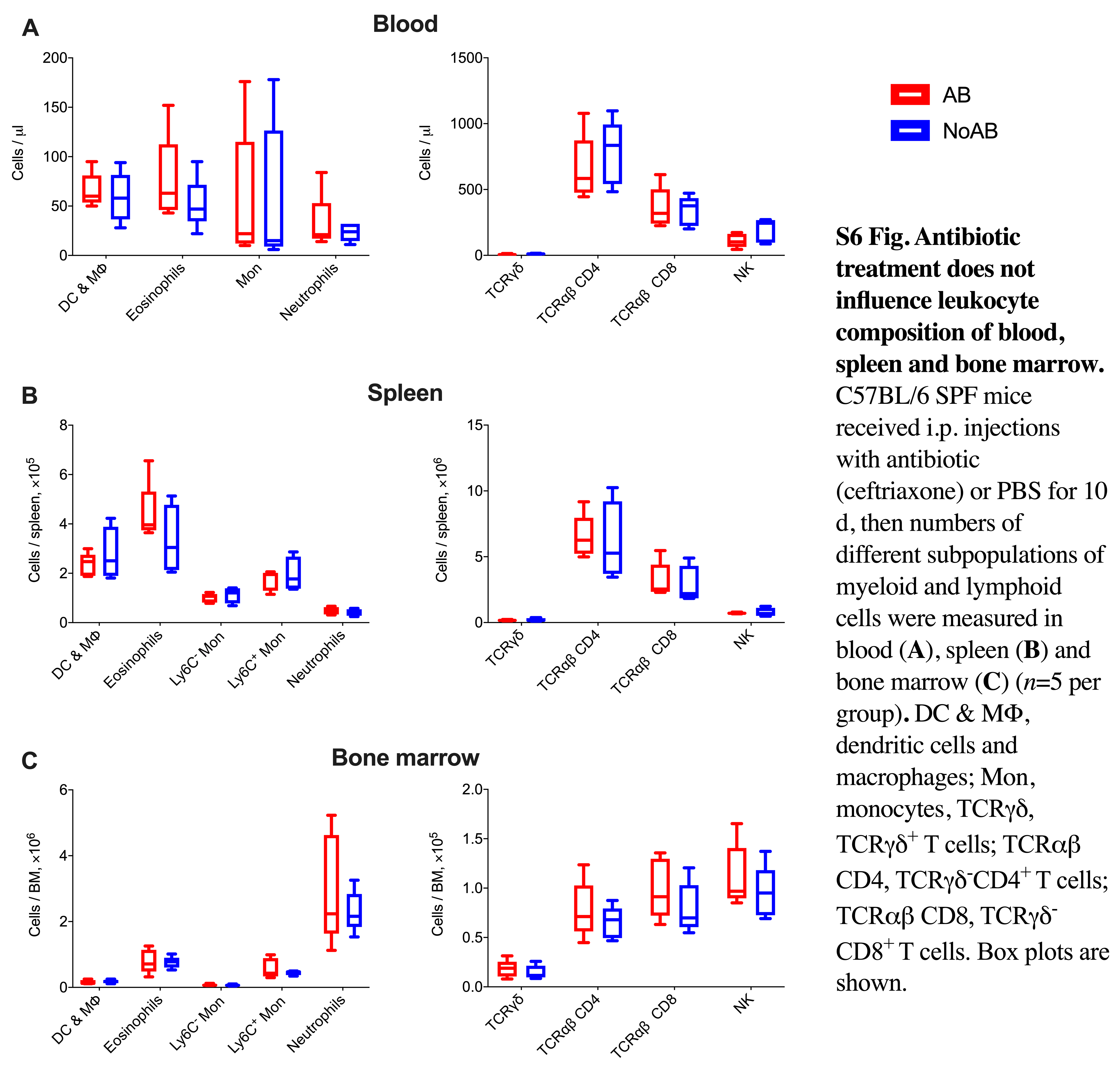

Supplement: S6 Fig — C57BL/6 SPF mice received i.p. injections with antibiotic (ceftriaxone) or PBS for 10 d, then numbers of different subpopulations of myeloid and lymphoid cells were measured in blood (A), spleen (B) and bone marrow (C) (n = 5 per group). DC & MΦ, dendritic cells and macrophages; Mon, monocytes, TCRγδ, TCRγδ+ T cells; TCRαβ CD4, TCRγδ-CD4+ T cells; TCRαβ CD8, TCRγδ-CD8+ T cells. Box plots are shown. (TIFF) [file ppat.1009854.s006.tiff]

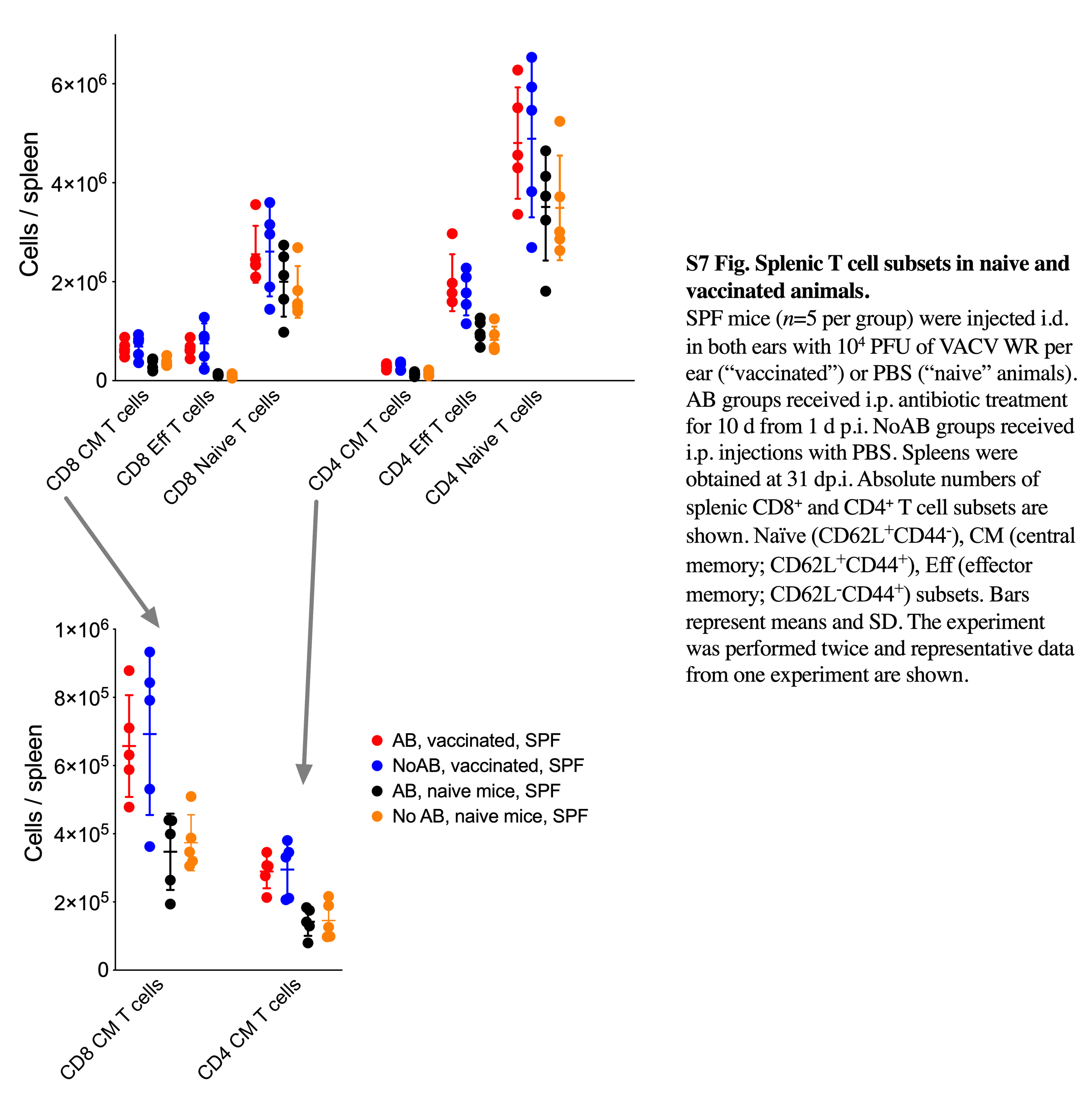

Supplement: S7 Fig — SPF mice (n = 5 per group) were injected i.d. in both ears with 104 PFU of VACV WR per ear (“vaccinated”) or PBS (“naive” animals). AB groups received i.p. antibiotic treatment for 10 d from 1 d p.i. NoAB groups received i.p. injections with PBS. Spleens were obtained at 31 d p.i. Absolute numbers of splenic CD8+ and CD4+ T cell subsets are shown. Naïve (CD62L+CD44-), CM (central memory; CD62L+CD44+), Eff (effector memory; CD62L-CD44+) subsets. Bars represent means and SD. The experiment was performed twice and representative data from one experiment are shown. (TIFF) [file ppat.1009854.s007.tiff]

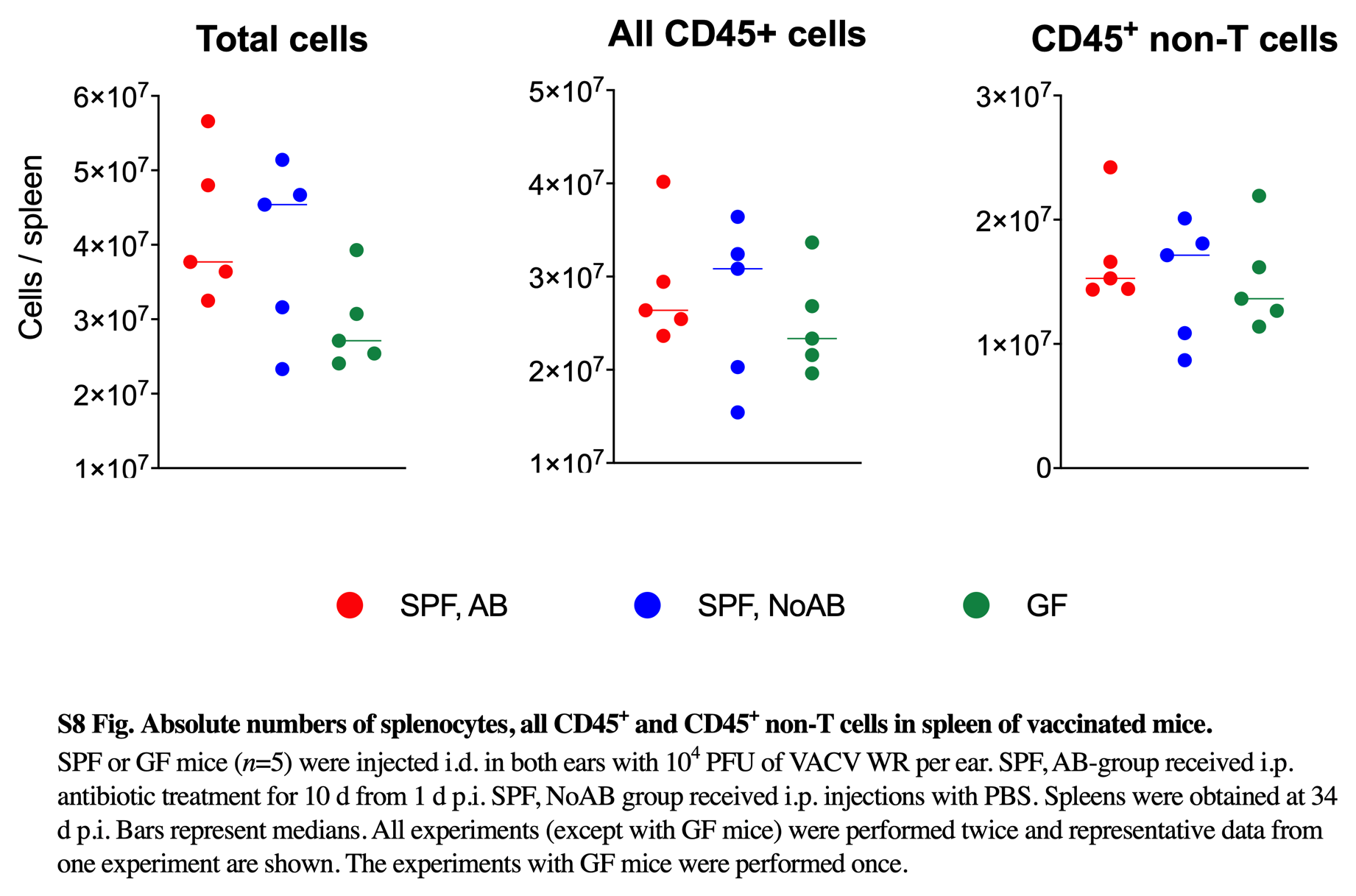

Supplement: S8 Fig — SPF or GF mice (n = 5) were injected i.d. in both ears with 104 PFU of VACV WR per ear. SPF, AB-group received i.p. antibiotic treatment for 10 d from 1 d p.i. SPF, NoAB group received i.p. injections with PBS. Spleens were obtained at 34 d p.i. Bars represent medians. All experiments (except for GF mice) were performed twice and representative data from one experiment are shown. The experiments with GF mice were performed once. (TIFF) [file ppat.1009854.s008.tiff]

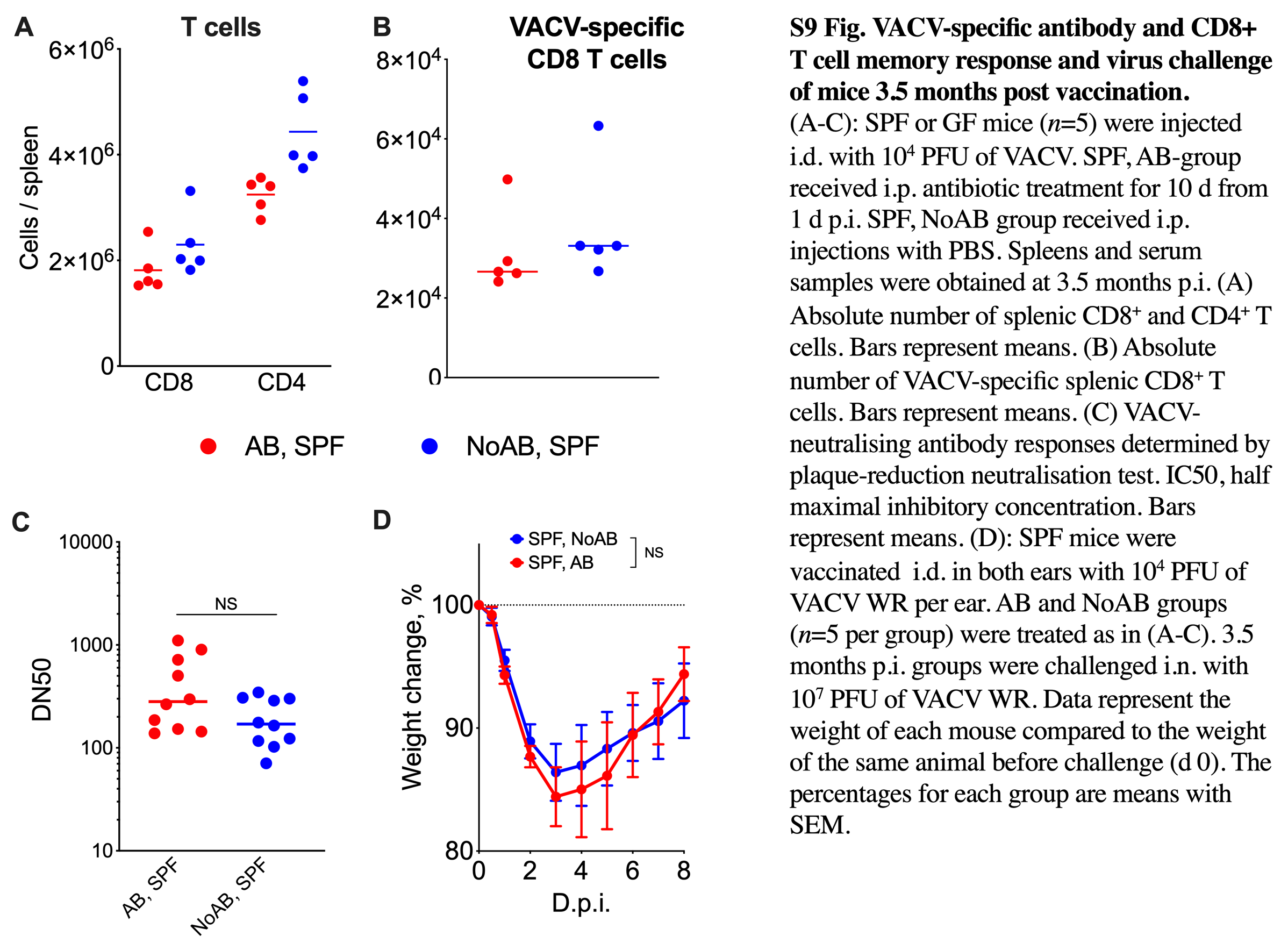

Supplement: S9 Fig — (A-C): SPF or GF mice (n = 5) were injected i.d. with 104 PFU of VACV. SPF, AB-group received i.p. antibiotic treatment for 10 d from 1 d p.i. SPF, NoAB group received i.p. injections with PBS. Spleens and serum samples were obtained at 3.5 months p.i. (A) Absolute number of splenic CD8+ and CD4+ T cells. Bars represent means. (B) Absolute number of VACV-specific splenic CD8+ T cells. Bars represent means. (C) VACV-neutralising antibody responses determined by plaque-reduction neutralisation test. IC50, half maximal inhibitory concentration. Bars represent means. (D): SPF mice were vaccinated i.d. in both ears with 104 PFU of VACV WR per ear. AB and NoAB groups (n = 5 per group) were treated as in (A-C). At 3.5 months p.i. groups were challenged i.n. with 107 PFU of VACV WR. Data represent the weight of each mouse compared to the weight of the same animal before challenge (d 0). The percentages for each group are means with SEM. (TIFF) [file ppat.1009854.s009.tiff]

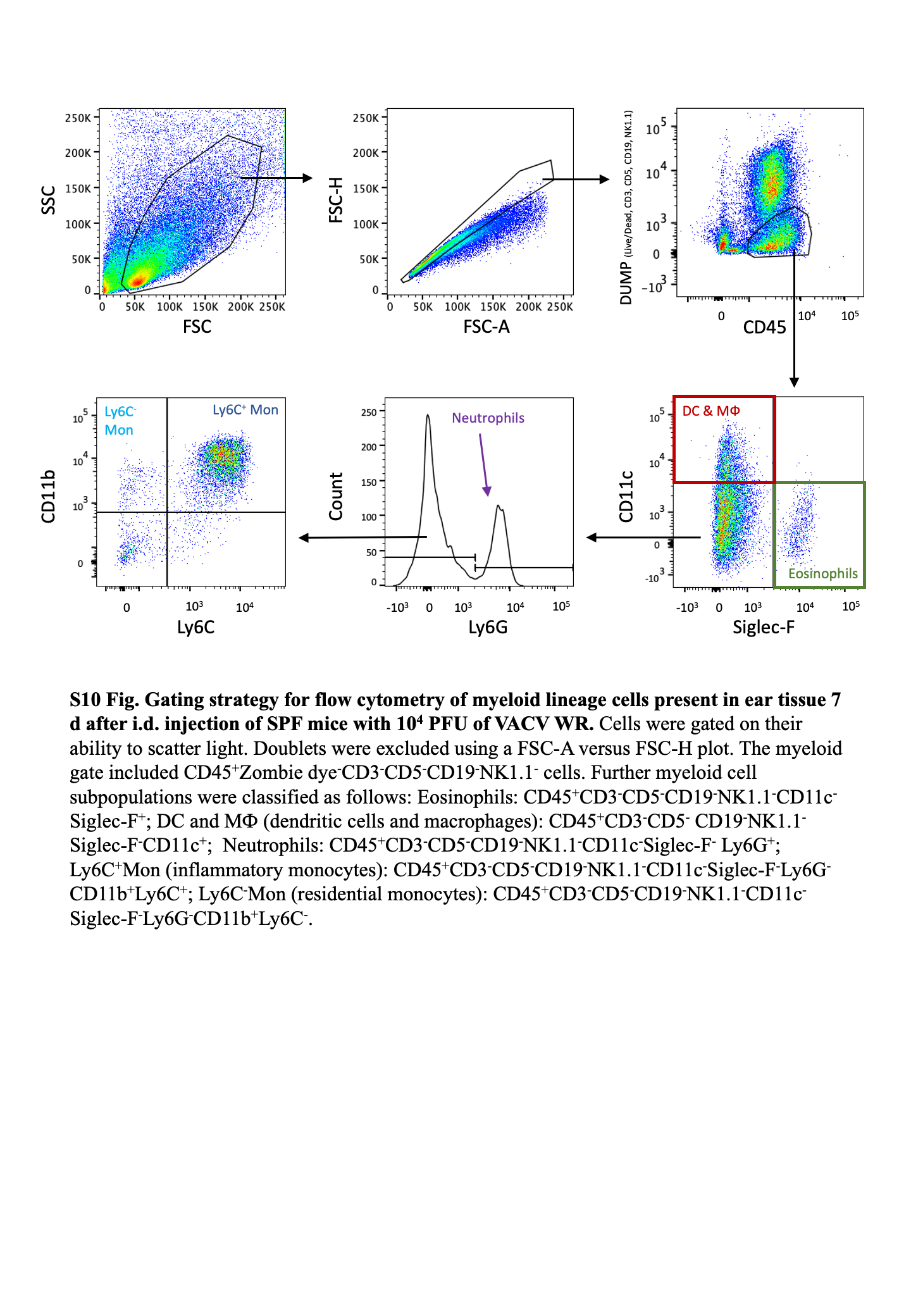

Supplement: S10 Fig — Cells were gated on their ability to scatter light. Doublets were excluded using a FSC-A versus FSC-H plot. The myeloid gate included CD45+Zombie dye-CD3-CD5-CD19-NK1.1- cells. Further myeloid cell subpopulations were classified as follows: Eosinophils: CD45+CD3-CD5-CD19-NK1.1-CD11c-Siglec-F+; DC and MΦ (dendritic cells and macrophages): CD45+CD3-CD5- CD19-NK1.1-Siglec-F-CD11c+; Neutrophils: CD45+CD3-CD5-CD19-NK1.1-CD11c-Siglec-F- Ly6G+; Ly6C+Mon (inflammatory monocytes): CD45+CD3-CD5-CD19-NK1.1-CD11c-Siglec-F-Ly6G-CD11b+Ly6C+; Ly6C-Mon (residential monocytes): CD45+CD3-CD5-CD19-NK1.1-CD11c-Siglec-F-Ly6G-CD11b+Ly6C-. (TIFF) [file ppat.1009854.s010.tiff]

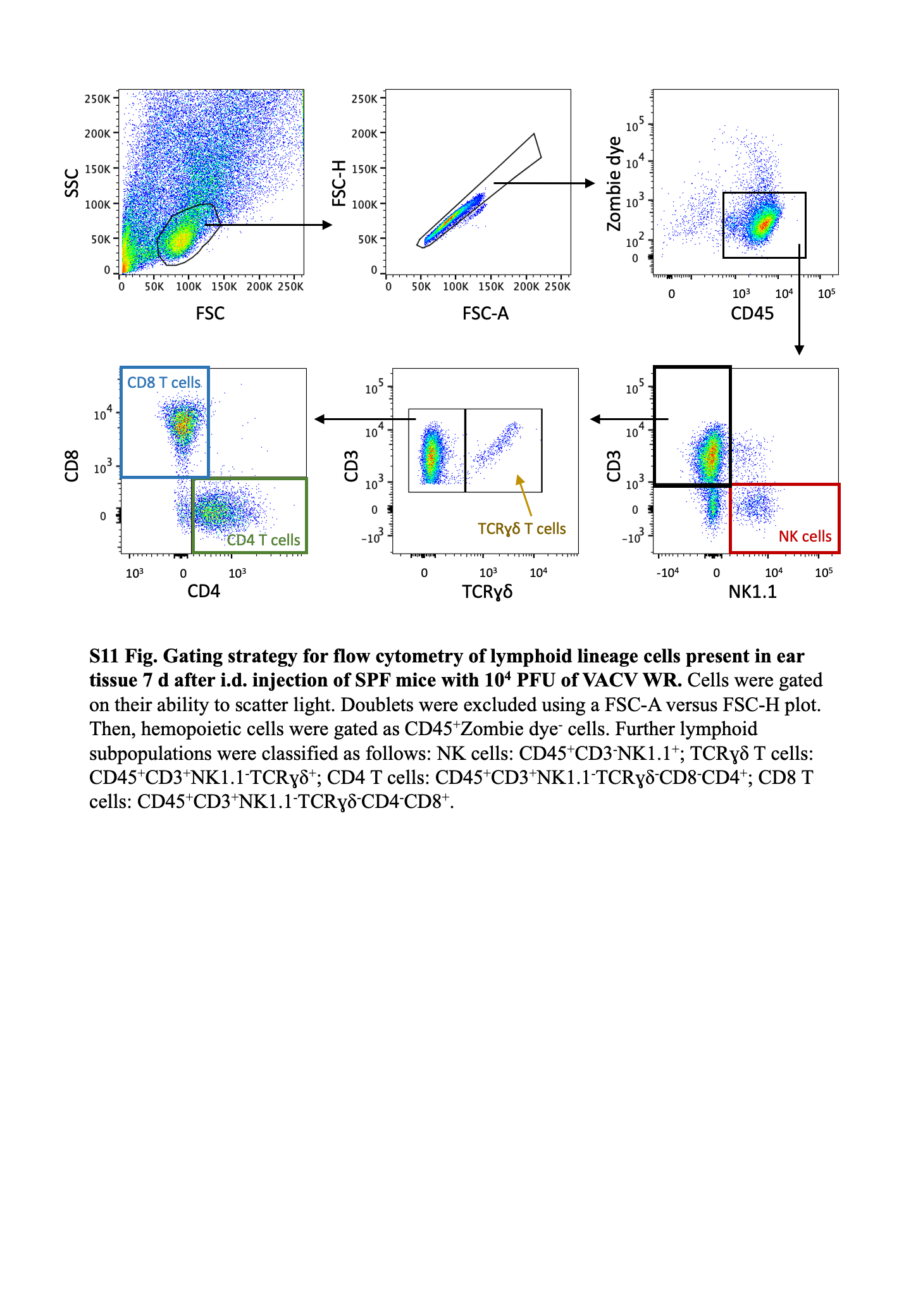

Supplement: S11 Fig — Cells were gated on their ability to scatter light. Doublets were excluded using a FSC-A versus FSC-H plot. Then, hemopoietic cells were gated as CD45+Zombie dye- cells. Further lymphoid subpopulations were classified as follows: NK cells: CD45+CD3-NK1.1+; TCRɣδ T cells: CD45+CD3+NK1.1-TCRɣδ+; CD4 T cells: CD45+CD3+NK1.1-TCRɣδ-CD8-CD4+; CD8 T cells: CD45+CD3+NK1.1-TCRɣδ-CD4-CD8+. (TIFF) [file ppat.1009854.s011.tiff]

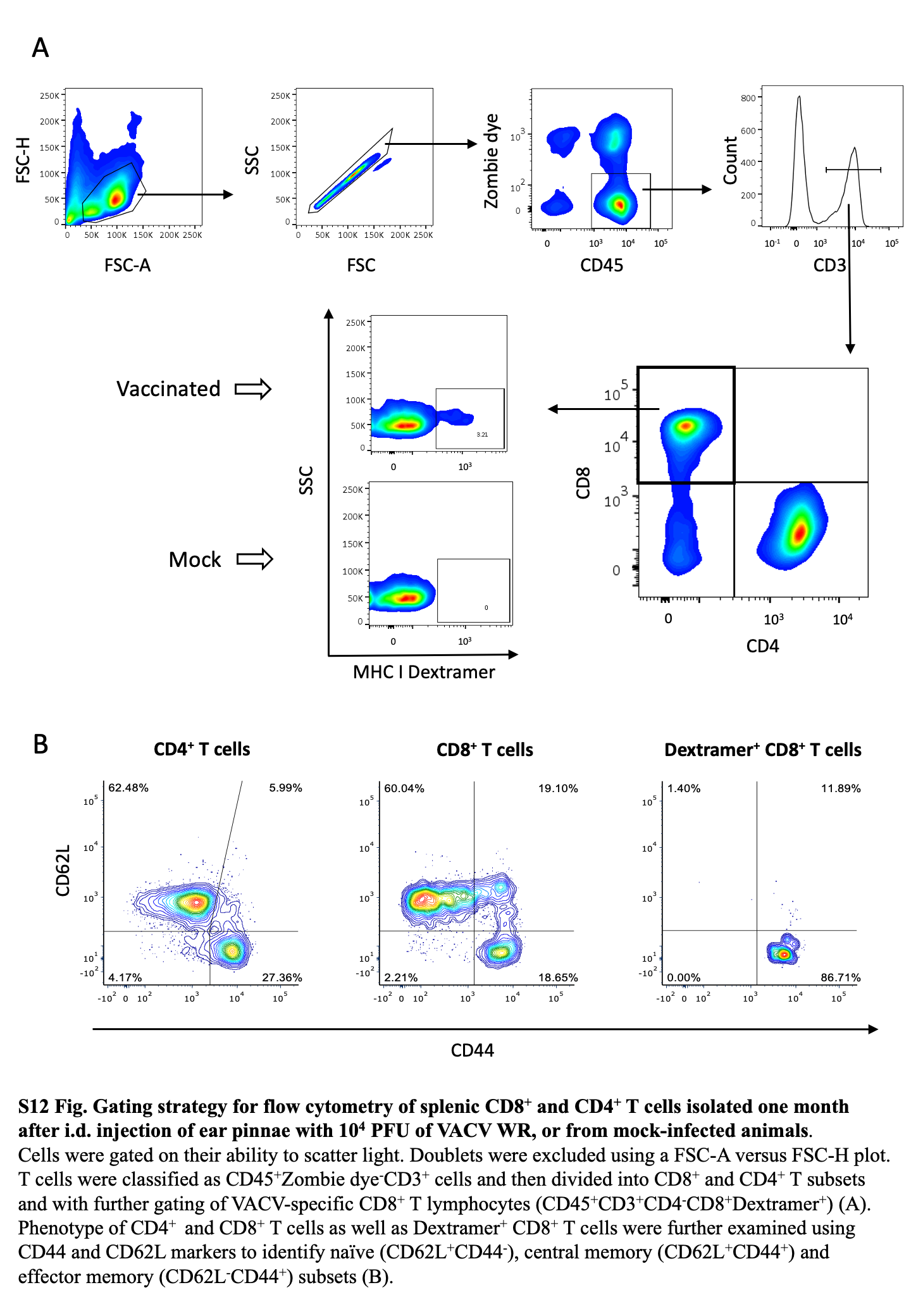

Supplement: S12 Fig — Cells were gated on their ability to scatter light. Doublets were excluded using a FSC-A versus FSC-H plot. T cells were classified as CD45+Zombie dye-CD3+ cells and then divided into CD8+ and CD4+ T subsets and with further gating of VACV-specific CD8+ T lymphocytes (CD45+CD3+CD4-CD8+Dextramer+) (A). Phenotype of CD4+ and CD8+ T cells as well as Dextramer+ CD8+ T cells were further examined using CD44 and CD62L markers to identify naïve (CD62L+CD44-), central memory (CD62L+CD44+) and effector memory (CD62L-CD44+) subsets (B). (TIFF) [file ppat.1009854.s012.tiff]
