# Supplementary material for: Smallpox vaccination induces a substantial increase in commensal skin bacteria that promote pathology and influence the host response
Source: PLoS Pathog. 2022 Apr 21;18(4):e1009854. doi: 10.1371/journal.ppat.1009854 (PMC9022886; doi:10.1371/journal.ppat.1009854)
Supplement: S1 Table — (PDF) [file ppat.1009854.s013.pdf]

S1 Table. Variation in bacterial composition (beta diversity) among groups of samples at different times p.i. as well as mock and intact ear specimens by PERMANOVA test with and without Bonferroni correction.

Taxon: Genus

Permutation N:	9999
Total sum of squares:	23.73
Within-group sum of squares:	20.74
F:	2.37
p (same):	0.0001

p values

	VACV, Day 2	VACV, Day 5	Mock	VACV, Day 8	VACV, Day 12
Intact	0.0121 *	0.0636	0.0001 *	0.0001 *	0.001 *
VACV, Day 2		0.4126	0.0012 *	0.0001 *	0.0032 *
VACV, Day 5			0.0378 *	0.0003 *	0.0084 *
Mock				0.0002 *	0.0077 *
VACV, Day 8					0.6891

Bonferroni corrected p values

	VACV, Day 2	VACV, Day 5	Mock	VACV, Day 8	VACV, Day 12
Intact	0.1815	0.954	0.0015 *	0.0015 *	0.015 *
VACV, Day 2		1	0.018 *	0.0015 *	0.048 *
VACV, Day 5			0.567	0.0045 *	0.126
Mock				0.003 *	0.1155
VACV, Day 8					1

Taxon: Species

Permutation N:	9999
Total sum of squares:	28.23
Within-group sum of squares:	24.52
F:	2.483
p (same):	0.0001

p values

	VACV, Day 2	VACV, Day 5	Mock	VACV, Day 8	VACV, Day 12
Intact	0.002 *	0.015 *	0.0001 *	0.0001 *	0.0002 *
VACV, Day 2		0.2924	0.001 *	0.0001 *	0.0003 *
VACV, Day 5			0.0484 *	0.0004 *	0.0036 *
Mock				0.0002 *	0.0013 *
VACV, Day 8					0.0597

Bonferroni corrected p values

	VACV, Day 2	VACV, Day 5	Mock	VACV, Day 8	VACV, Day 12
Intact	0.03 *	0.225	0.0015 *	0.0015 *	0.003 *
VACV, Day 2		1	0.015 *	0.0015 *	0.0045 *
VACV, Day 5			0.726	0.006 *	0.054
Mock				0.003 *	0.0195 *
VACV, Day 8					0.8955

P values between different groups of samples. p < 0.05 marked with *.
