# Supplementary material for: Smallpox vaccination induces a substantial increase in commensal skin bacteria that promote pathology and influence the host response
Source: PLoS Pathog. 2022 Apr 21;18(4):e1009854. doi: 10.1371/journal.ppat.1009854 (PMC9022886; doi:10.1371/journal.ppat.1009854)
Supplement: S2 Table — (PDF) [file ppat.1009854.s014.pdf]

S2 Table. Monoclonal antibodies and dyes used for staining cells prior to analysis by flow cytometry.

Antibody/dye	Clone	Source
CD3-APC	145-2C11	553066, BD Biosciences
CD3-BV421	145-2C11	562600, BD Biosciences
CD4-APC-H7	GK1.5	560181, BD Biosciences
CD4-BUV395	GK1.5	563790, BD Biosciences
CD5-BV421	53-7.3	562739, BD Biosciences
CD8-APC-R700	53-6.7	564983, BD Biosciences
CD8-BV605	53-6.7	100744, BioLegend
CD11b-APC-R700	M1/70	564985, BD Biosciences
CD11b-PE	M1/70	101208, BioLegend
CD11c-BV650	N418	117339, BioLegend
CD19-BV421	1D3	562701, BD Biosciences
CD44-BB515	IM7	564587, BD Biosciences
CD45-PerCP	30-F11	557235, BD Biosciences
CD45-PE	30-F11	103106, BioLegend
CD62L-APC-Cy7	MEL-14	104428, BioLegend
Ly6C-BV510	HK1.4	128033, BioLegend
Ly6C-APC	HK1.4	128016, BioLegend
Ly6G-APC-H7	1A8	565369, BD Biosciences
MHC Dextramer H-2Kb/TSYKFESV/PE		JD3267-PE, Immudex
NK1.1-BV421	PK136	562921, BD Biosciences
NK1.1-BV650	PK136	564143, BD Biosciences
Siglec-F-BB515	E50-2440	564514, BD Biosciences
γδ T-Cell Receptor-BV510	GL3	563218, BD Biosciences
Zombie Violet Fixable Viability Kit		423113, BioLegend
