# Supplementary material for: Smallpox vaccination induces a substantial increase in commensal skin bacteria that promote pathology and influence the host response
Source: PLoS Pathog. 2022 Apr 21;18(4):e1009854. doi: 10.1371/journal.ppat.1009854 (PMC9022886; doi:10.1371/journal.ppat.1009854)
Supplement: S1 Methods — (DOCX) [file ppat.1009854.s015.docx]

**S1 Methods**

# Neutrophils isolation and microscopic examination

To confirm that neutrophils identified by FACS analysis had morphological characteristics appropriate for neutrophils, the cells were isolated from ear tissues at 9 d p.i. with VACV and stained with Zombie Fixable Viability dye, anti-CD3, CD5, CD19, NK1.1, CD11c, CD45, Siglec-F, Ly6G mAbs (S1 Table). Cells were then resuspended in 4% PFA/PBS. Cell sorting was carried out by MoFlo (Beckman Coulter); Zombie dye^-^CD3^-^CD5^-^CD19^-^NK1.1^-^CD11c^-^CD45^+^Siglec-F^-^Ly6G^+^cells were sorted on glass slides. Mowiol mounting medium containing 1 µg/ml of DAPI were added to mount the glass coverslip. Slides were examined under a Zeiss LSM700 confocal microscope (Carl Zeiss AG). Images were analysed with ZEN Black (Carl Zeiss AG) and ImageJ (NIH Image) software.

# Trucount FACS assay

Blood was collected into Micro K3EDTA Tubes (Sarstedt, Cat. # 41.1395.005) to prevent clot formation. Then, 50 µl of whole blood was pipetted into the bottoms of BD Trucount Tubes (BD Biosciences, Cat. # 340334), followed by 5 min incubation with Mouse BD Fc Block. The samples were then stained with mAbs to CD45, CD3, CD4, CD8, CD19, CD11b, CD11c, NK1.1, TCRɣ𝛿, Siglec-F, Ly6C and Ly6G (S1Table). After lysis of red blood cells, and without washing steps, the numbers of different leukocyte populations were determined by analysis on a BD LSRFortessa.

# Processing and analysis of 16S sequence data

# Bacterial 16S rRNA gene analysis

The forward and reverse fastq files of each sample were processed according to the MOTHUR MiSeq manual with some modifications (MOTHUR wiki at <http://www.mothur.org/wiki/MiSeq_SOP>). The "make.contigs" command was used with no extra parameters. All ambiguous sequences (maxambig=0) and sequences containing homopolymers longer than 8 bp (maxhomop=8) were removed with the “screen.seqs”. The quality-screened sequences were aligned using the Silva bacterial database "silva.nr_v123.align" with flip parameter set to true. Any sequences outside the expected alignment coordinates were also removed using the "screen.seqs" command. The alignment coordinates were set with "optimise=start-end, criteria=90". The correct aligned sequences were filtered using the "filter.seqs" command with "vertical=T" and "trump=.". The subsequent filtered sequences were de-noised by allowing three mismatches in the "pre.clustering" step and chimaeras were removed using Uchime with the dereplicate option set to "true". The chimaera-free sequences were classified using the Silva reference database "silva.nr_v123.align" and the Silva taxonomy database "silva.nr_v123.tax" and a cut off value of 80%. The “classify.seqs” step reported the present of eukaryotic, mitochondrial and archaeal sequences that were subsequently removed with the “remove.lineage command. The majority of reads were of cellular (predominantly mitochondrial) origin, as tissue samples were used (rather than swabs from the skin surface) to identify bacteria, present within skin lesions and on the skin surface. The high quality, chimaera-free, and correct classified sequences were normalised using the "sub.sample" command. Each sample was normalised to a maximum of 10000 reads. Subsequently, the normalised samples were used for oligotyping.

An alternative independent analysis of sequence data was made as follows. Raw FASTQ files were obtained from paired-end sequencing (75 nt) on Illumina MiSeq. The DADA2 package (BioConductor) was used to filter and trim sequences using a probabilistic noise model [1]. After filtering the sequences and removing the chimeræ, these data were compared to the SILVA database of bacteria and labelled. Sequences were assembled into groups called ASVs (Amplicon Sequence Variants) [2] instead of the traditional OTUs (Operational Taxonomic Units). The Phyloseq package was used (R/BioConductor) for statistical analysis of ASVs.

# Oligotyping and taxa identification

Oligotyping was used for clustering the high quality filtered fasta sequences from the MOTHUR pipeline. Oligotyping is a computational method to investigate the diversity of closely related by distinct bacterial organisms in final operational taxonomic units identified in environmental data sets through 16S ribosomal RNA gene data by the canonical approaches. For oligotyping we used the “Minimum Entropy Decomposition” (MED) option for sensitive partitioning of high-throughput marker gene sequences from the oligotyping pipeline [3]. The normalised high quality fasta and name file from MOTHUR were renamed by appending the group name to the sequence name, using the "rename.seqs" command. Then, a redundant renamed-fasta file was generated using the "deunique.seqs" command, which creates a redundant fasta file from a fasta and name file. The redundant fasta file was then used for oligotyping using the unsupervised "Minimum Entropy Decomposition" (MED) for sensitive partitioning of high-throughput marker gene sequences [3]. The command line was "decompose fasta.file --gen-html –g –t -" using default parameters accept the –t character which was set to a dash "-" character. The dash character was used in MOTHUR "rename.seqs" command to separate the sample name from the unique info in the define of the sequence name.

MED was conducted with a minimum substantive abundance of an oligotype setting of 10 (-M parameters was set to 10). This setting removed 31,500 outliers (4.5%) from a total of 699,368 sequences and generated 3236 final nodes (oligotypes). The oligotype representative sequences were classified using the Ribosomal Database Project web portal (RDP release 11, Update 5, September 30, 2016). The “strain” was set to type, the “source” was set to isolates, the “quality” was set to good, and the “taxonomy” was set to nomenclatural. A total of 841 redundant species were identified. When we removed the contaminant, species identified in the 6 blank controls and the two positive controls, we were left with 811 species.

We generated a total of 26,651,694 sequences of which 2,028,740 sequences were high quality, chimaera free, mitochondrial free and correct aligned sequences to the bacterial variable region 4 in the Silva v123 bacterial 16S database. We used a maximum of 10,000 high quality sequences per sample (min 990, max 10000, average 7435) which were used for oligotyping.

A total of 166 families were classified with a relative abundance between 17.8% and 0.0018%. Of these 12 families had a minimum of 1% abundance in all samples which contained 77.83% of all reads, and 63 families had a minimum of 0.1% abundance in all samples that contained 96.9% of all reads.

A total of 417 genera were classified with a relative abundance between 17.7% and 0.0013%. Of these, 18 genera had a minimum of 1% abundance in all samples which contained 67.78% of all reads and 85 genera had a minimum of 0.1% abundance in all samples that contained 93.37% of all reads.

A total of 811 species were classified with a relative abundance between 15.64% and 0.00108%. Of these, 17 species had a minimum of 1% abundance in all samples that contained 52.61% of all reads and 124 species had a minimum of 0.1% abundance in all samples that contained 84.18% of all reads.

# Contamination detection in bacterial 16S samples

A very stringent in-house pipeline was followed to remove environmental and laboratory contaminants. The methods used to reduce contamination included using sequencing appropriate controls, reducing contamination as much as possible during collection and extraction by using sterile materials and applying general aseptic techniques, and randomising sample handling as much as logistically possible. Once the sequencing was performed, contaminant oligotypes were identified using the negative and blank controls. In a second step we used Spearman rho’s correlation coefficient analysis to correlate obvious contaminants with suspicious contaminants. At the end we removed ten contaminating species which were *Acinetobacter haemolyticus, Pseudomonas lurida, Amaricoccus macauensis, Propionibacterium acnes, Thermogutta hypogea, Yersinia enterocolitica, Aquabacter spiritensis, Escherichia/Shigella coli, Cloacibacterium rupense, and Pseudomonas psychrophile*. The contaminants made up 14% of all reads.

# Heatmap analysis of the microbiome data

The heatmap was done using the R package Heatplus version 2.28.0.

The “cuth” parameter i.e. the height at which to cut through the dendrogram to define groups (clusters) of similar feature was set such that five clusters were identified. These five clusters are colour coded with red, blue, green, violet, and orange.

The taxon abundance data were clustered using the “vegdist” function from the VEGAN package with the Bray method and then the columns and rows were clustered using the default “hclust” function from the stats package with ward.D method.

For the heatmaps, we used all samples with a minimum abundance of 1% in at least 5 samples. The heatmaps show 61 genera from the genus taxon datasets.

# Beta diversity analysis

Multivariate beta diversity analysis was performed using PERmutational Multivariate Analysis Of VAriance (PERMANOVA). The similarity index was set to Bray-Curtis and for the Permutation N: we used the default of 9999. For the pairwise PERMANOVA test we reported the Bonferroni adjusted P value and the F statistic value. The PERMANOVA test was done using the PAST3 statistical software package, version 3.20 [4].

**References**

Callahan BJ, McMurdie PJ, Rosen MJ, Han AW, Johnson AJ, Holmes SP. DADA2: High-resolution sample inference from Illumina amplicon data. Nat Methods. 2016;13: 581-583.

Callahan BJ, McMurdie PJ, Holmes SP. Exact sequence variants should replace operational taxonomic units in marker-gene data analysis. ISME J. 2017; 11: 2639-2643.

Eren AM, Morrison HG, Lescault PJ, Reveillaud J, Vineis JH, Sogin ML. Minimum entropy decomposition: unsupervised oligotyping for sensitive partitioning of high-throughput marker gene sequences. ISME J. 2015; 9: 968-979.

Hammer Ø, Harper DAT, Ryan PD. PAST: Paleontological statistics sofware package for education and data analysis. Palaeontologia Electronica. 2001;4: 1-9.
